# Response to Ayurvedic therapy in the treatment of migraine without aura

**DOI:** 10.4103/0974-7788.59941

**Published:** 2010

**Authors:** Prakash Balendu Vaidya, Babu S. R. Vaidya, Sureshkumar K. Vaidya

**Affiliations:** *V C P Cancer Research Foundation (SIRO), Turner Road, Clement Town, Dehradun, India*; 1 *Padaav, 13^th^ Main Lakkasandra Extension, Bangalore, India*; 2 *Padaav, 180 D, 12th Main, 11^th^ Cross Saraswathipuram, Mysore, India*

**Keywords:** Alternative therapy, Ayurveda, CAM, migraine

## Abstract

Migraine patients who do not respond to conventional therapy, develop unacceptable side-effects, or are reluctant to take medicines resort to complementary and alternative medicines (CAM). Globally, patients have been seeking various non-conventional modes of therapy for the management of their headaches. An Ayurvedic Treatment Protocol (AyTP) comprising five Ayurvedic medicines, namely Narikel Lavan, Sootshekhar Rasa, Sitopaladi Churna, Rason Vati and Godanti Mishran along with regulated diet and lifestyle modifications such as minimum 8 h sleep, 30-60 min morning or evening walk and abstention from smoking/drinking, was tried for migraine treatment. The duration of the therapy was 90 days. Out of 406 migraine patients who were offered this AyTP, 204 patients completed 90 days of treatment. Complete disappearance of headache and associated symptoms at completion of AyTP was observed in 72 (35.2%); mild episode of headache without need of any conventional medicines in 72 (35.2%); low intensity of pain along with conventional medicines in 50 (24.5%); no improvement in seven (3.4%) and worst pain was noted in three (1.4%) patients, respectively. In 144 (70.5%) of patients marked reduction of migraine frequency and pain intensity observed may be because of the AyTP. Though the uncontrolled open-label design of this study does not allow us to draw a definite conclusion, from this observational study we can make a preliminary assessment regarding the effectiveness of this ayurvedic treatment protocol.

## INTRODUCTION

Migraine is a widespread, chronic and intermittently disabling disorder characterized by recurrent headaches with or without aura.[1] Recent studies estimate the prevalence of migraine at about 6-8% in men and 12-15% in women. In terms of actual numbers of attacks, combined figures from prevalence and incidence studies suggest 3000 migraine attacks occur every day for each million of the general population.[2] The rate of migraine varies globally, and although there is a lack of epidemiological data available in many countries at present, recent anecdotal evidence suggests higher rates in certain places like India.[3]

Pharmaceutical treatment of migraine is complex, with no agreed upon guidelines. Most individuals often need medication during acute attacks and some prophylactic measure to reduce attacks.[2] Moreover, the uncertainty regarding treatment and the need to perhaps trial patients on a variety of drugs adds to the escalating costs. Some specific drugs such as Triptans and ergotamine tartrate are often expensive and not commonly used in resource-poor countries, resulting in a significant amount of pain and disability.[4] Another problem is the actual overuse of such medications which causes ‘medication overuse headache’ (MOH), further complicating management strategies.[5]

A large percentage of patients do not respond to pharmacological interventions for migraine headache, develop unacceptable side-effects, or are reluctant to take medications[6]. As a result many patients resort to many complementary and alternative therapies like acupuncture,[7] biofeedback therapy,[8] relaxation therapy, herbal remedies and vitamin or mineral supplementation.[6] Recent studies have demonstrated the effectiveness of acupuncture[9] and Yoga[10] in the reduction of migraine headache. The use of complementary and alternative medicine (CAM) in migraine is a growing phenomenon which, though increasingly widespread, is poorly understood.[11] Ayurveda is a traditional medical system used by a majority of India's 1.1 billion population.[12] The principal author in his clinical practice first observed that a judicious combination of five Ayurvedic medicines can markedly reduced the migraine frequency in some migraine patients. Later, an Ayurvedic Treatment Protocol (AyTP) comprising these five ayurvedic medicines along with regulated diet and lifestyle modifications was developed for migraine treatment. This AyTP was tried by over 600 migraine patients.[13] The background work of this AyTP was first carried out in Dhanwamtari Ayurvedic College and Hospital, Chandigarh from June 2002 to December 2004. [13] In this series we report the analysis of the observational prospective clinical study of this AyTP carried on 406 migraine patients in nine major cities of south India.

## MATERIALS AND METHODS

### Study period

May 2005 to March 2007.

### Settings

This study was carried out in the clinics of 17 Vaidyas in nine major cities of South India, the details of which are given in Table 1.

**Table 1:** Study carried out in the clinics of 17 Vaidyas in nine major cities of South India

Place	Name of Vaidya	Patients Enrolled
Aurangabad	Vd. N Patil	7
Bengaluru	Vd. SS Hiremath, Vd. S Kulkarni, Vd. VM	264
(Bangalore)	Bhat Aroor, Vd. Prashanth MV, Vd, R Babu	
Bellary	Vd. Shailaja HB, Vd. Hema Desai DN, Vd. Savitha BP	37
Dharwad	Vd. Sushma RH	10
Hyderabad	Vd. Sangamesh Benne, Vd. Vijaysimha R	12
Mysore	Vd. Prasanna Venkatesh TS, Vd. K Suresh Kumar	31
Shimoga	Vd. Chitralekha V Krishna	8
Tumkur	Vd. Sunil Kumar K	31
Warangal	Vd. Mallikarjun K	6

### Treatment Protocol

The treatment protocol was derived from the Ayurvedic concept of diagnosis of *Amla-Pitta* a state of acid-alkali imbalance causing one of the symptoms of *Shiro ruja* (headache). A uniform AyTP was developed which comprised of a combination of five ayurvedic formulations (Narikel Lavan (NL), Sootshekhar Rasa (SR), Sitopaladi Churna (SC), Rason Vati (RV) and Godanti Mishran (GM)[14,15] along with regulated diet (three meals and three snacks providing adequate calories and meals devoid of nicotine, caffeine, reheated food, aerated drink), and lifestyle modification included minimum 8 h sleep, moderate exercise such as morning or evening walk for 30-60 min and abstention from smoking / drinking.

### Composition

#### Narikela Lavan

Narikel shell - *Cocus nucifera*

Saindhava lavana - Rock salt

A fully-ripe coconut is taken, the shell is removed and a hole is made at the top of the coconut. Powdered rock salt is put through the hole till the water in the coconut rises to the level of the hole. The coconut is then covered by clay smeared cloth in three consecutive layers and dried. This is put into a puta of 10-15 cowdung cakes/ furnace. When cool, the charred coconut containing salt is powdered in a khalva.

The composition of other medicines is given in Tables 2–5.

**Table 2:** Composition of Sootshekhar Rasa

Traditional name	English / scientific name	Proportion
Suddha Parada	Processed cinnabar	1 part
Suddha Gandhaka	Processed sulphur	1 part
Dalchini	*Cinnamomum zeylanica*	1 part
Chhoti Elachi	*Elleteria cardamomum*	1 part
Tej patta	*Cinnamomum tamala*	1 part
Nagkesar	*Mesua jerrea*	1 part
Shankh Bhasma	*Turbinella pyrum*	1 part
Swarna makshika Bhasma	Chalco pyrite	1 part
Ropya Bhasma	Argentum	1 part
Tamra Bhasma	Cuprum	1 part
Dhatura's seed	*Datura metel*	1 part
Suhaga	Borax sodium borate	1 part
Saunti	*Zingiber officinale*	1 part
Kali mircha	*Piper nigrum*	1 part
Chhoti pippal	*Piper longum*	1 part
Bhringraj swarasa	*Eclipta Alba*	Q.S (for mardana)

**Table 3:** Composition of Sitopaladi Churna

Traditional name	English / scientific name	Proportion
Mishri	Sugar candy	16 parts
Vanslochan	*Bambusa arumdimaceo*	8 parts
Chhoti Pippali	*Piper longum*	4 parts
Chhoti Elachi	*Ellettaria cardamomum*	2 parts
Dalchini	*Cinnamomum zeylanica*	1 part

**Table 4:** Composition of Rason Vati

Traditional name	English / scientific name	Proportion
Lasuna	*Allium sativum*	1 part
Jiraka	*Cuminum cyminum*	1 part
Saindhava lavana	Rock salt	1 part
Gandhaka-suddha	Processed sulphur	1 part
Sunthi	*Zingiber officinale*	1 part
Marica	*Piper nigrum*	1 part
Pippali	*Piper longum*	1 part
Hingu	*Ferula foetida*	1 part
Nimbu rasa	*Citrus medica* juice	QS for bhavana

**Table 5:** Composition of Godanti Mishran

Traditional name	English / scientific name	Proportion
Godanti bhasma	Gypsum	8 parts
Jahar mohara pisti	Serpentine	2 parts
Rasadi vati		2 parts

### Medicine dosage and duration

The daily recommended doses of these combined formulations were 7.3 g per day (NL 2000 mg, SR 375 mg, SC 1425 mg, RV 3000 mg, GM 500 mg). The treatment period was for 90 days. During AyTP the patients were not allowed to take any other alternative medications.

### Manufacturing of Medicine

The Ayurvedic medicines were prepared by Bharat Bhaishajaya Shala Private Limited, Dehradun under the manufacturing license issued by the Government of Uttarakhand, India. The medicines are manufactured under the GMP guidelines and following the stringent procedures as mentioned in the classical texts of Ayurveda.

### Subjects and Diagnosis

A total of 406 migraine patients (M:133; F:273) were offered this AyTP. All the patients were chronic sufferers and had earlier consulted a neurologist for their migraine.

### Eligibility

Criteria for inclusion were: individuals of either gender, age 10 and above, meeting the International Classification of Headache Disorders (ICHD) criteria [16] for migraine without aura [Table 6]. Exclusion criteria included marked depression, anxiety or psychosis; more than two visits/month for mental healthcare; more than one psychiatric medication; major medical illness under treatment; pregnancy.

**Table 6:** Eligibility of subjects for observational clinical study on AyTP for migraine

**Inclusion Criteria**	**Exclusion Criteria**
Subject > 10 years of age	Marked depression, anxiety or
Either gender	psychosis
Meet ICHD* criteria for migraine	Major medical illness under
Headache history > 2 years	treatment
Willing to follow the dietary	Pregnancy
restriction	Clotting disorders
Willing to complete daily diary	More than 2 visits/month for
Willing to take the medication	mental healthcar
for 90 days	Use of any other alternative
	medication during AyTP study
	period

* International Classification of Headache Disorders

### Screening, consent, and enrollment

Interested migraine patients who contacted Vaidyas in their clinics were first screened for eligibility. The patients then underwent a baseline medical assessment, including a complete medical history and physical examination. The abdomen of all patients was especially examined. Patients having any serious health problem or comorbid illness were not selected for undergoing AyTP. Eligible patients were first explained the treatment procedure in detail. Patients who were willing to follow the set norms of the AyTP were then enrolled for the study after taking a written consent. Clinical details of the patients were entered in a clinical record form (CRF), which was updated after each visit.

### Monitoring Progress of Patients

Eligible patients were instructed to maintain a daily headache diary after the start of AyTP. Subjects had the option of completing the diary either on paper / postcard and mailing it to their respective Vaidyas. Alternatively, they could report via telephone. However, all patients were asked to visit the clinics at the start of therapy, then at Day 30, 60 and at stoppage of therapy at Day 90. However, patients were advised to report to the clinic in case of emergency.

### Outcome Measures

The primary outcome variables for this study included frequency and intensity of headache and self-perceived benefit of the intervention at Day 30, 60 and 90. Detailed description of these variables follows:

#### Headache frequency

Determination from the daily headache diary the total number of headache frequency the patient had after the start of therapy. The subjects were also instructed to record the presence and intensity of their headaches on a daily basis. Additionally, the subjects were invited to comment on the nature of their headache, the associated symptoms, and the suspected triggers.

#### Headache intensity

The visual analogue scale (VAS) and numeric rating scale (NRS) was used to measure the intensity of pain. VAS from no pain (= 0) to worst pain imaginable [=10 (or 100)] and the five- point categorical verbal rating scale (VRS) i.e., score 0 = none; 1-3 = mild; 4-6 = moderate; 7-8 = severe; 9-10 = worst. Guide to grading headache intensity was included with each diary.

#### Headache-related disability: MIDAS

Disability, defined as the consequences of illness on the ability to work and function, is measured using the Migraine Disability Assessment Score (MIDAS).[17] Derived from the Headache Impact Test, MIDAS is a seven-item questionnaire that assesses the number of days during the previous three months that respondents missed work or school, experienced decreased productivity at work or home, or missed social engagements because of headaches. The definition of various grades is mentioned in Table 7.

**Table 7:** The definition of various grades

Grade	Definition	Score
I	Minimal or infrequent disability	0-5
II	Mild or infrequent disability	6-10
III	Moderate disability	11-20
IV	Severe disability	21+

### Adverse events

Reports of adverse events were obtained from the patients during their visit to the clinic, self-reports in the headache diaries or by direct contact with patients via telephone.

### Statistical Analysis

Kruskal Wallis test was used to compare the VAS score on day 30, day 60 and day 90 from the base line. Paired t-test was used to compare the headache days. The level of significance was set at *P*<0.05. The statistical analysis was performed using the Statistical Package for Social Sciences (SPSS 12.0).

## RESULT

**Figure 1:**
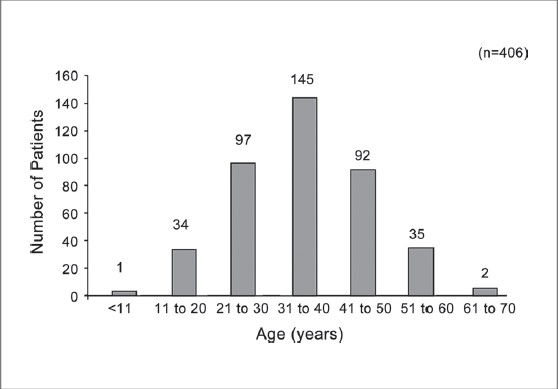
Agewise distribution of migraine patients

**Figure 2:**
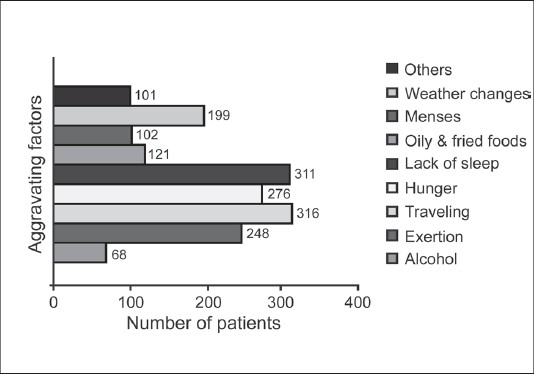
Various aggravating factors reported by migraine patients

**Figure 3:**
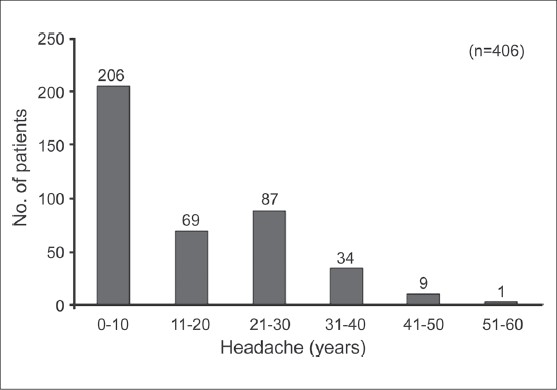
History of headache of migraine patients

**Figure 4:**
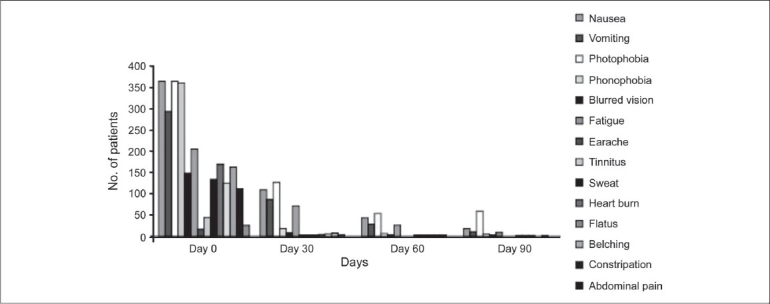
Various associated symptoms with migraine

The prevalence of migraine was found to be higher in the age group 20-50 years, with the highest ranging between >30-< 40 years [Figure 1]. Around 90% of the patients were non-vegetarian and 155 (38%) patients had family history of headache. Details of the prior treatment of migraine patients indicated that 231 (57%) patients were totally dependent on allopathic medicine; 167 (41%) patients had tried both allopathic and alternative medicine such as Homeopathy, Unani / Siddha, Ayurveda and Naturopathy etc., and eight (2%) patients were totally dependent on alternative medicine. It was found that exertion, lack of sleep and hunger were the three most important factors for aggravating migraine, and details of other factors are given in Figure 2. History of headache of migraine patients ranged from 1 to 60 years [Figure 3]. At the time of enrollment all the patients reported more than five attacks in a year from occasional, daily, alternate day and five to eight days in a month. The maximum patients those who were enrolled had migraine attack once a week. Maximum migraineurs complained of nausea, photo phobia, phono phobia, and vomiting as associated symptoms [Figure 4]. Nearly 50% reported moderate to extreme fatigue besides heartburn, belching, blurred vision, flatus, constipation etc.

**Figure 5:**
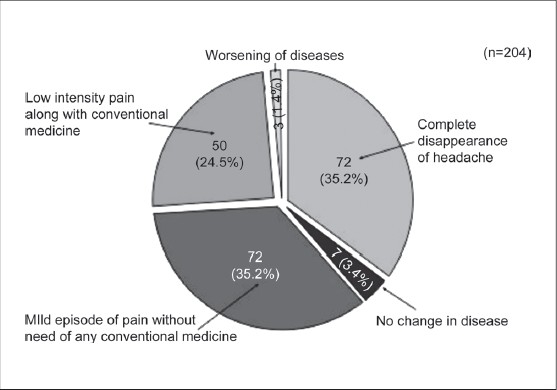
Overall response of Ayurvedic treatment protocol on completion of 90 days of therapy

A total of 406 patients were offered this AyTP, however, after 30 days 129 (31.7%) and after 60 days 41(14.8%) patients dropped out, respectively. A total of 204 (50.2%) patients completed 90 days of therapy. Complete disappearance of headache and associated symptoms at completion of AyTP was observed in 72 (35.2%); mild episode of headache without need of any conventional medicines in 72 (35.2%); low intensity of pain along with conventional medicines in 50 (24.5%); no improvement in seven (3.4%) and worst pain was noted in three (1.4%) patients, respectively [Figure 5]. There was a significant reduction in the VAS score after the start of AyTP [Table 8].

**Table 8:** Effect of AyTP on VAS score of migraine patients

VAS score	N	Mean	SD	P value
Day 0		356	0.72	----
Day 30	204	2.15	1.11	<0.001
Day 60		1.40	1.08	<0.001
Day 90		1.0	0.93	<0.001

**Figure 6:**
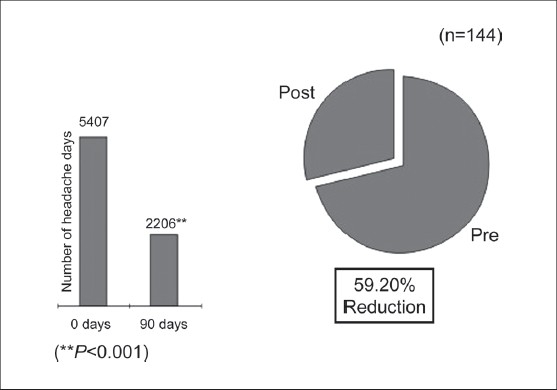
Impact of AyTP on days of occurrence of headache

To study the impact of the AyTP on headache days 166 patients were randomly interviewed at the time of enrollment and after completing 90 days of treatment. It was observed that there was a highly significant reduction in headache days among the treated migraineurs [Figure 6]. Decrease in MIDAS score was observed after 90 days of therapy. At the start of therapy the number of patients assigned various grades of MIDAS were as follows: Grade I -110 (27.0%); Grade II - 70 (17.2%); Grade III-100 (24.6%); Grade IV-126 (31.0%), which changed after the completion of therapy viz. Grade I-171 (83.8%); Grade II -18 (8.8%); Grade III-nine (4.4%), and Grade IV-four (1.9%), respectively.

In 45 (11.0%) patients it was observed that the clinical condition deteriorated after the start of AyTP, hence, the therapy was stopped immediately. However, patients who responded to this therapy and completed 90 days of treatment did not show any noticeable side-effects.

## DISCUSSION

Though Ayurvedic therapy is popular among migraine sufferers, there are very few reports available on the efficacy and toxicity of these therapies. Moreover, classical Vaidyas treat patients on the basis of presenting symptoms and hence, there is quite a variation in the selection of Ayurvedic medicines by different Vaidyas. However, for the present study a uniform treatment protocol (AyTP) was first designed and the same was offered to all migraine patients. Generally, the patients who visited the clinics for AyTP were not satisfied with conventional therapy. Many wanted to try the AyTP on an experimental basis. The first author in his clinical practice observed that the combination of five Ayurvedic therapy can reduce the migraine frequency and intensity of pain in some patients and hence further collected sufficient evidence to start a clinical trial on 131 migraine patients in the Dhanwamtari Ayurvedic College and Hospital, Chandigarh in 2002.[15] The results of the trial encouraged us to take up the present investigation in a planned manner. This AyTP is now being used by various Vaidyas in various places of India, especially in the southern parts.

Migraine was distinguished from common headache by Tissot in 1783 for the first time who ascribed it to a supra-orbital neuralgia provoked by reflexes from the stomach, gall bladder or uterus. Later, migraine was classified as a neurological disorder. Our hypothesis is quite similar to Tissot's idea on the pathogenesis of migraine, viz. that it usually arose from stomach disturbance.[18] Incidentally, there is a close correlation between the symptoms of migraine with those of *Amla-pitta* (state of acid-alkali imbalance in the body) causing symptoms such as: *brahma* (confusion), *moorcha* (fainting), *aruchi* (anorexia), *aalasya* (fatigue), *chardi* (vomiting), *prasek* (nausea), mukhmadhurya (sweetness in the mouth) and shiroruja (headache). The correlation between the cause and symptoms of *Amla-pitta* match the current diagnosis criteria of migraine. It may be possible that the combination of Narikel Lavan, Sootashekhara Rasa, Sitopaladi Churna, Rason Vati and Godanti Mishran in conjunction with regulated lifestyle and diet may have restored the acid-alkali balance, and restored / strengthened the functioning of the gastrointestinal system. A better acid-alkali balance in the body may have been responsible for reducing the frequency of migraine.

The herbo-mineral Ayurvedic medicines used for the migraine treatment contained *Bhasma*[19] of silver, copper and mercury and many immunomodulatory medicinal herbs, namely *Alium sativum*, *Eclipta alba*, *Cinnmomum zeylanica*, *Zingiber officinalis*, *Piper longum, Piper nigrum, Bambusa arumdinaceae*, *Ellettaria cardamomum* and *Cinnamomum cassia*, *Ferula northrax*, *Citrus acida* etc. Some ingredients used for medicine preparations are moderate to severely toxic in the raw form (ashodhit). However, intrigue processing (shodhan) converts these toxic materials to complex mineral forms which are nontoxic. However, improper processing/ manufacturing of Ayurvedic medicines may result in severe toxicity.[20] Hence, the safety profile of the combined formulations was first established in animal models. It was observed that mice feed four times and rats 10 times more the daily equivalent human dose did not produce any toxicity.[21] In this present study it was observed that in 11% of patients the clinical conditions deteriorated after the start of the AyTP. We are at this moment unable to explain what was the exact cause for this; however, the Ayurvedic medicine was well tolerated by other patients.

During the last few decades, plants have been increasingly employed as a herbal remedy for migraine treatment and prophylaxis,[22] examples include Feverfew (*Tanacetum parthenium*),[23] Butterbur (*Petasites hybridus*),[24], etc. Most surveys agree that herbal remedies are amongst the most prevalent therapies and that headache/migraine is one of the most frequent reasons for trying plant-derived medications.[25] Complimentary and Alternative Medicine (CAM) is often perceived by the public to be more helpful than conventional care for the treatment of headache.[26] Recent studies have indicated that Ayurvedic medicines can be effective in treatment of tension-type headache.[27]

From this observational study we can make a preliminary assessment regarding the effectiveness of this Ayurvedic treatment protocol in migraine treatment. Baring a few, the Ayurvedic medications were well tolerated by patients. Marked reduction of migraine frequency and pain intensity observed in patients with AyTP needs attention. However, to ascertain the real effectiveness of this AyTP a properly controlled clinical trial with a larger patient population is required.
